# Supplementary material for: Beneficial Effects of Milk-Derived Extracellular Vesicles on Liver Fibrosis Progression by Inhibiting Hepatic Stellate Cell Activation
Source: Nutrients. 2022 Sep 29;14(19):4049. doi: 10.3390/nu14194049 (PMC9571732; doi:10.3390/nu14194049)
Supplement: Supplementary file 1 [file nutrients-14-04049-s001.zip › S2 Primer sequences.pdf]

**Primer sequences**		
**Gene name**	**Forward primer**	**Reverse primer**
GAPDH mouse	AATCCCATCACCATCTTC	GGCAGTGATGGCATGGA
α-SMA mouse	AGCCAGTCGCTGTCAGGAA	CGAAGCCGGCCTTACAGA
COL1A1 mouse	TGTGTTCCCTACTCAGCCGTCT	CTCGCTTCCGTACTCGAACG
TIMP1 mouse	TCTGCAACTCGGACCTGGTC	TCCCACAGCCTTGAATCCTT

miRNA qPCR primers obtained from Quantabio (Beverly, MA, USA).

miR-148a-3p (HSMIR-0148A-3P)
miR-29 (HSMIR-0029), miR-21 (HSLET-0021)
Let-7a (HSLET-0007A-5P)
RNU6 (HS-RNU6)
