# Supplementary figures and images for: Beneficial Effects of Milk-Derived Extracellular Vesicles on Liver Fibrosis Progression by Inhibiting Hepatic Stellate Cell Activation
Source: Nutrients. 2022 Sep 29;14(19):4049. doi: 10.3390/nu14194049 (PMC9571732; doi:10.3390/nu14194049)

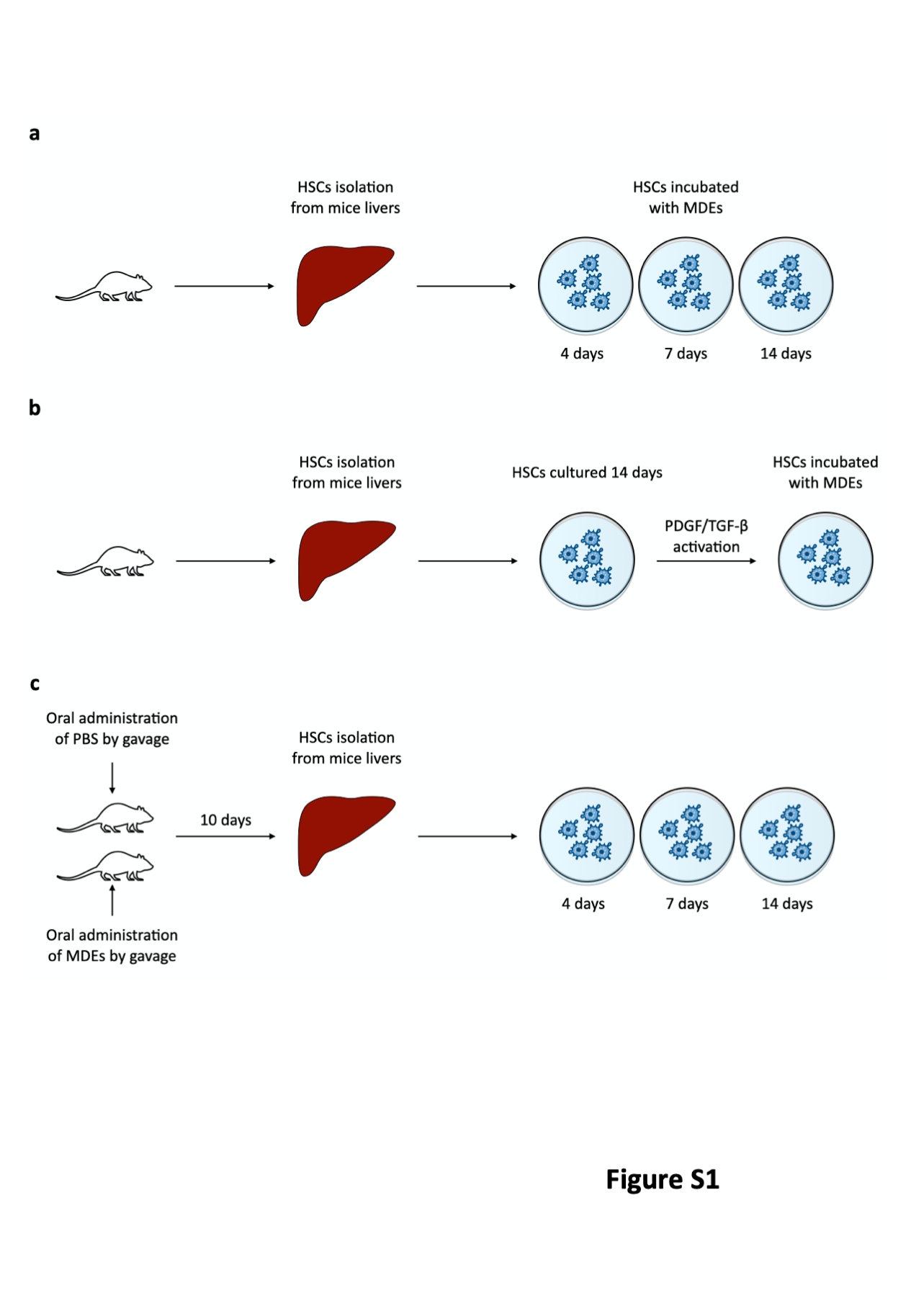

Supplement: Supplementary file 1 [file nutrients-14-04049-s001.zip › S1.tiff]

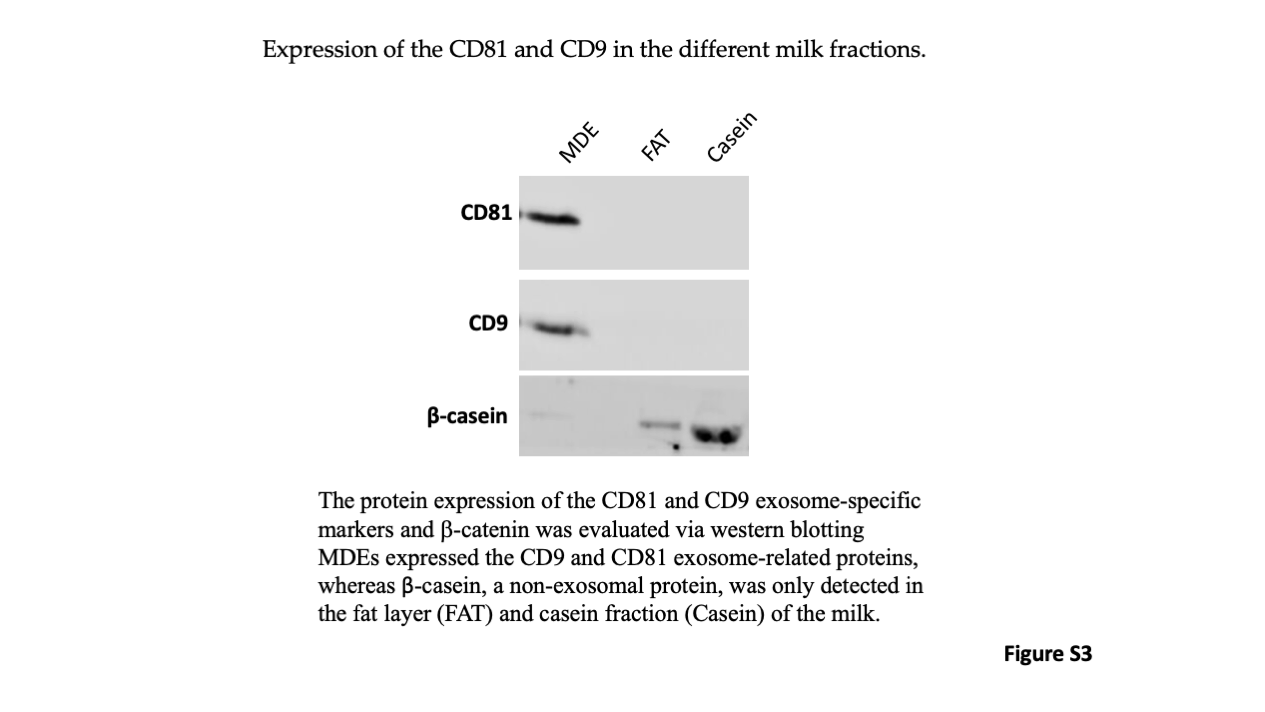

Supplement: Supplementary file 1 [file nutrients-14-04049-s001.zip › S3.tiff]

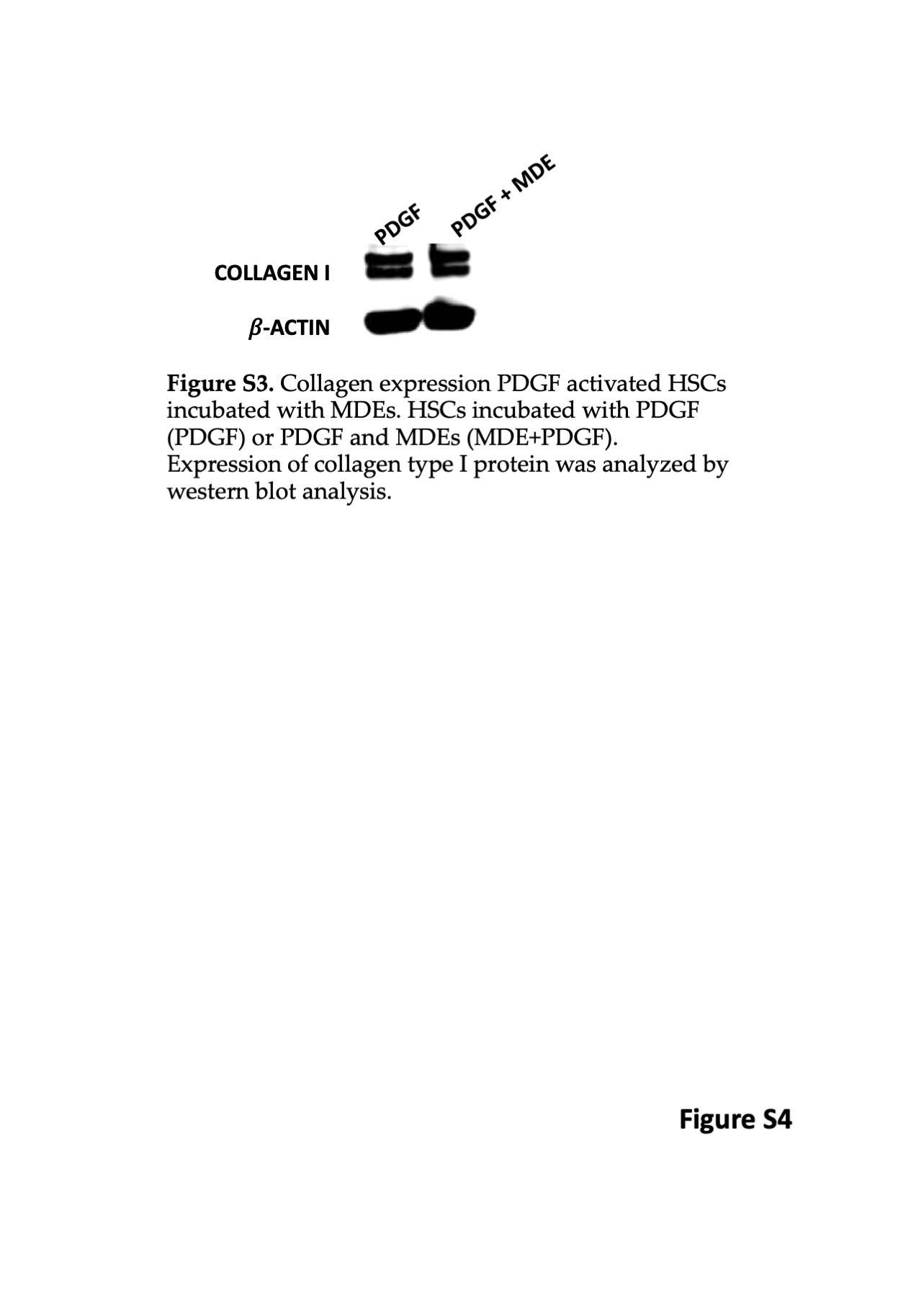

Supplement: Supplementary file 1 [file nutrients-14-04049-s001.zip › S4.tiff]
